# Lectures on Animal Heat

**Published:** 1845-05

**Authors:** 


### BIBLIOGRAPHICAL NOTICES.

*Lectures on Animal Heat.* By Thomas Spencer, M.D., Professor of the Institutes and Practice of Medicine in the Medical Institution of Geneva College. 12mo., pp. 114. Geneva, N. Y., 1845.

This little book comprises the lectures of Prof. Spencer on animal heat, published at the request of his class. In endeavouring to answer the question, what is the source of animal heat, he has discussed the kindred subjects of nutrition and sanguification. In the brief notice which we are able to present of his performance, we cannot enter into a particular examination of his alleged facts and reasonings, in the order in which he has presented them, but merely propose to give our readers a general idea of the contents of the book.

In explaining his views, Dr. Spencer has not been backward in appealing to chemical affinities, acting under the control of the vital forces. Indeed, in this respect, he has raised a much higher superstructure of speculation than Liebig himself, and upon a much narrower foundation of facts. He conceives that the dark colour of venous blood depends upon a pigment, consisting of carbon, hydrogen, and oxygen, which, as he supposes it to contain hydrogen and oxygen in the proportion to form water, he calls *hydrate of carbon.* The theory which maintains that a combination of hydrogen and carbon, called *hydro-carbon,* is thrown off from the blood in the lungs, is objected to by the author, because it is an imaginary compound; but we would ask, is not the author's hydrate of carbon an imaginary compound? It is true that Dr. Spencer contends that his hydrate of carbon is analogous to, if not identical with, the lignin of vegetables; yet not intended, like lignin, to be deposited as an element of growth, but destined to be eliminated, as an excrementitious substance, by the lungs and skin. If these considerations are sufficient to make the "hydrate of carbon" a real and not a hypothetical substance, reasons not less cogent would make the "hydro-carbon" of blood a real compound; since compounds of the same elements are known to exist out of the body.

Dr. Spencer not only imagines that a compound of the nature of lignin exists in the venous flood, but exists there in the solid state! To show that we have not misunderstood him, we give his own words:—"You may say, from what has been advanced, that these dense atoms of hydrate of carbon, being the components of woody fibre, must be infinitesimal blocks of wood, floating in the sanguineous mass. This is, doubtless, true." p. 31.

All the theories of animal heat of any importance suppose the oxidation of carbon, or of carbon and hydrogen to be its source. Where this oxidation takes place, and under what peculiar circumstances, are the chief questions to be answered. In order to explain why heat is given out when carbon is converted into carbonic acid, and when gases and liquids are solidified, Dr. Spencer goes into some explanations in relation to the capacities of bodies for heat. On this subject our knowledge is very imperfect. In the case of the conversion of carbon into carbonic acid, it is not necessary to prove a diminution of capacity in the product compared with the capacity of the oxygen consumed, before we can assume that caloric is extricated. It is sufficient to know that, during the quick oxidation (combustion) of carbon out of the body, much heat is given out, and to feel assured from chemical laws, that a similar oxidation, by gaseous oxygen, cannot take place within the body, without the emission of the same quantity of heat. It is a reasonable theory that gaseous substances, weight for weight, contain a larger quantity of caloric than the liquids or solids from which they are formed; yet matter, when gasefied by the energy of chemical forces, does not always absorb an additional amount of caloric, or, in other words, produce cold. Thus, when we pour muriatic acid on chalk, we get a large quantity of carbonic acid gas, derived from the same acid in the solid state, and yet cold is not produced. The result of such experiments seems to show that certain gases, when condensed by chemical combination, carry with them, in their condensed state, all the caloric which they had previously contained.

The theory, formerly most prevalent in regard to calorification, was that animal heat had its source in the lungs. The theory of the day is, that it is generated in the systemic capillaries, during the conversion of arterial into venous blood. Dr. Spencer supposes animal heat to be extricated in both these localities. In the systemic capillaries, he assumes that his hydrate of carbon is formed from water and from the carbon of carbonic acid; and as he views the hydrate to be a solid, floating in the venous blood, he considers its formation as necessarily attended with the extrication of caloric, because in it water and the carbon of carbonic acid are solidified. After the hydrate of carbon arrives at the lungs as a constituent of the venous blood, it undergoes what is equivalent to combustion. The hydrogen present, being saturated with oxygen, does not burn; but the carbon forms carbonic acid with the oxygen of the air in respiration, and generates in the lungs sufficient heat, not only to vaporize the water of the hydrate of carbon, but to be extricated in a free state. As the hydrate of carbon, by the assumption made, obtains its carbon from carbonic acid, the author, at first, is somewhat puzzled to know what to do with the oxygen of the acid. Finally, he disposes of it by uniting it with the protoxide of iron in the blood. We have here a number of postulates;—first, there is formed a hydrate of carbon during the conversion of arterial into venous blood; secondly, it is a solid, though the blood in which it is alleged to exist as a solid, is, throughout, a perfect liquid; thirdly, its formation causes an extrication of heat, because it is a solid formed from a liquid and a gas. Now, this same carbon, which, being condensed from carbonic acid, helps to produce free heat in the systemic capillaries, causes a fresh extrication of heat, by combining back again with oxygen, in the pulmonary capillaries. Here, it appears to us that too much is taken for granted. Can it be admitted, in the legitimate sense of the word solid, that solid matter floats in living blood as it circulates in the veins?

The speculations of Dr. Spencer are illustrated and enforced by a number of diagrams, in which the elements are denoted by their appropriate symbols. In them, the author has occasionally mixed up the notation of Berzelius with that of Liebig and Poggendorff, by sometimes denoting degrees of oxidation by dots, at other times by the symbol O.

We have marked several passages in these Lectures which contain errors, or statements of doubtful authority. At p. 2, the obsolete statement is made that atmospheric air contains one per cent. of carbonic acid; whereas, on an average, it contains only about one part in two thousand. We have nowhere met with the statement made by Dr. Spencer, that Dalton estimated the latent heat of oxygen to be *four times* as great as that of carbonic acid gas. Neither do we believe that rain water contains common salt; although a passage in Liebig's Agricultural Chemistry seems to support this opinion.

This book is disfigured by a number of typographical errors, which may be excused on account of the hasty manner in which, to answer the wishes of the Professor's class, it was necessary to send the sheets to press. (*Preface.*) The paper is good, and the type clear and distinct, merits which much enhance the pleasure of reading a book.

Upon the whole, we rise from the perusal of Dr. Spencer's book, with the impression that he has shown much ingenuity in his attempt to explain several of the intricate functions of the vital organism. If the author has speculated too much, let us hope that he will hereafter atone for the error, by endeavouring to add to our stock of physiological facts.

